# Evolutionary Algorithm for Improving Decision Tree with Global Discretization in Manufacturing

**DOI:** 10.3390/s21082849

**Published:** 2021-04-18

**Authors:** Sungbum Jun

**Affiliations:** Department of Industrial and Systems Engineering, Dongguk University, Seoul 04620, Korea; sbjun@dgu.ac.kr

**Keywords:** fault detection, interpretability, decision tree, evolutionary algorithm, discretization

## Abstract

Due to the recent advance in the industrial Internet of Things (IoT) in manufacturing, the vast amount of data from sensors has triggered the need for leveraging such big data for fault detection. In particular, interpretable machine learning techniques, such as tree-based algorithms, have drawn attention to the need to implement reliable manufacturing systems, and identify the root causes of faults. However, despite the high interpretability of decision trees, tree-based models make a trade-off between accuracy and interpretability. In order to improve the tree’s performance while maintaining its interpretability, an evolutionary algorithm for discretization of multiple attributes, called Decision tree Improved by Multiple sPLits with Evolutionary algorithm for Discretization (DIMPLED), is proposed. The experimental results with two real-world datasets from sensors showed that the decision tree improved by DIMPLED outperformed the performances of single-decision-tree models (C4.5 and CART) that are widely used in practice, and it proved competitive compared to the ensemble methods, which have multiple decision trees. Even though the ensemble methods could produce slightly better performances, the proposed DIMPLED has a more interpretable structure, while maintaining an appropriate performance level.

## 1. Introduction

Due to recent advances in Internet of Things (IoT), the connectivity between machines as well as the amount of data from sensors have been significantly increased. Depending on the need to leverage data properly, transparent and interpretable machine learning (ML) techniques are drawing particular attention amid growing interest in more reliable systems for a digital twin [1]. In particular, in fault detection of the manufacturing process (e.g., semiconductor manufacturing), interpretable ML models can provide insights into which attributes are the root causes for faults on the shop floor, so that human operators can improve the product quality [2].

However, even though various ML techniques having black-box structures (e.g., neural network) have been studied and developed for more accurate fault detection, many manufacturing companies suffer from the opaqueness of models, and costly human efforts to enhance the interpretability of detection accordingly [3]. In this context, the interpretability of ML models in manufacturing environments is growing in importance for two reasons. First, human operators want a set of understandable rules to control parameters in the manufacturing process. In addition, ML models with interpretability enable practitioners to explain the factors that have affected the ups and downs of process quality based on past production data [4].

Despite the fact that there is a significant need for interpretability, one of the most widely used interpretable models, decision-tree-based algorithms, still present some issues. The first issue is the trade-off between accuracy and interpretability. In order to improve the accuracy for training data, the maximum tree height should be increased. However, as the tree becomes deeper, the interpretability of the model decreases, because of the more complicated structure. Therefore, interpretable models are preferably small in size, as well as of sufficient high-performance. In order to have high explanation complexity, there is a significant need for shrinkage methods for ML models [5]. For example, a decision tree of depth = 5 is easier to understand than one of depth = 50.

However, when limiting the tree height for interpretability, many decision-tree-based algorithms, such as classification and regression tree (CART) and C4.5, allow trees to have only binary splits for continuous attributes, and thus hinder the potential for improving the performance of the decision trees within the limited tree depth.

To deal with these issues, discretization techniques for multi-point splits (decision tree algorithms) have been proposed [6,7]. The discretization techniques enable the information obtained from datasets to be more concise, easy to understand, and easy to use [8]. Nonetheless, efficient discretization techniques considering dependencies among attributes while maintaining interpretability have been far less studied even though the dependencies are important in performance [9]. In particular, for a decision tree algorithm, discretization of all continuous attributes without consideration of those dependencies has been shown to result in decreased accuracy [10].

In order to construct interpretable and effective models for fault detection, there is a significant need for efficient discretization algorithms designed for decision trees while considering dependencies between continuous attributes. Therefore, this paper proposes a novel approach for retrieving an improved decision tree for fault detection in manufacturing. The proposed approach utilizes the evolutionary process with *k*-means clustering to find good solutions efficiently for global discretization. In addition, to maintain high interpretability, the proposed approach is designed to improve a decision tree under the limitation of the maximum tree depth.

This paper is organized as follows. In Section 2, the previous research related to interpretable ML and discretization techniques is reviewed. Section 3 proposes a new evolutionary algorithm for discretization of continuous attributes based on *k*-means clustering. Section 4 defines the two datasets for fault detection in manufacturing, and Section 5 summarizes the results of experiments verifying the algorithm. Finally, Section 6 draws conclusions, and discusses possible areas for further research.

## 2. Backgrounds

### 2.1. Fault Detection in Manufacturing

Accurate detection of faults in manufacturing has been highly involved in the development of prediction models using data collected by sensors on the shop floor. Especially, well-designed ML models for fault detection at an early process can prevent defectives in the downstream, and thus significantly reduce manufacturing costs [11]. However, due to the interactions between process variables in large-scale manufacturing processes (e.g., chemical plant, semiconductor factory), identification of the relationships between fault causes and their effects is complicated [12].

While ML algorithms, such as neural network (NN) and support vector machine (SVM), have demonstrated high accuracy on several datasets, there is a significant issue called the “black-box” nature of their decision-making and learning processes. Because the learning process of black-box algorithms is neither transparent nor understandable to human operators, high accuracy on a given dataset may be misreading without a deeper understanding of causes from machine-related sensor inputs [13]. Therefore, interpretable ML-based models that can identify and analyze the root causes of fault detection in manufacturing have drawn attention from researchers.

### 2.2. Interpretable Machine Learning

**Figure 1:**
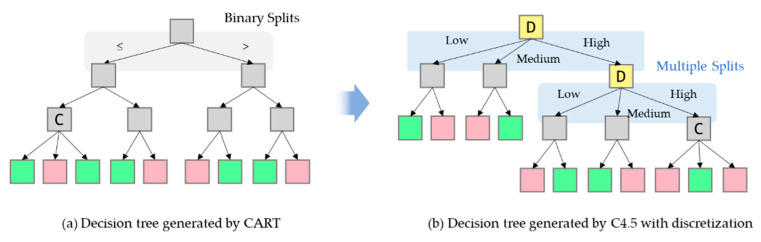
Illustration of a decision tree generated by CART and another tree generated by C4.5 with discretization. The discretized attributes are highlighted in yellow.

In order to deal with the black-box issue, interpretable ML models that are able to produce insights about their decisions have been investigated [14]. Among ML algorithms, decision-tree-based algorithms, such as ID3 (Iterative Dichotomiser 3) and C4.5 proposed by Quinlan [15,16], have been widely studied, due to their comprehensible nature that resembles the human reasoning process [17]. ID3 builds a decision tree for the given data in a top-down fashion and one categorical attribute is tested at each node. C4.5 is the successor to ID3 and it relaxes the restriction of ID3 that all attributes must be categorical. In addition, CART algorithm proposed by Reference [18] can address the classification and regression problems by creating a decision tree with binary splits of the continuous attributes as shown in Figure 1a. Compared to CART, C4.5 have an advantage of handling both continuous and discrete attributes [19]. These single-tree-based algorithms are easy to understand, and the trees can be easily converted to a set of rules. However, in spite of the high interpretability, the algorithms also have some limitations, such as overfitting, low performance, and binary splits on continuous attributes [20].

In order to overcome the limitations, various ensemble methods have been proposed and broadly investigated. Ensemble methods are simple and powerful techniques that aggregate predictions of some weak learners (such as decision trees) to provide more accurate estimation, instead of finding a single sophisticated learner [21]. For example, random forest introduced by Reference [22] generates many decision trees and aggregates their result. Especially, random forest has been shown to outperform other learners for problems with high-dimensional data.

In addition, boosting is a technique of combining a set of weak classifiers into one strong classifier for high-performance prediction, and has been a very successful technique for solving the two-class classification problem. The first practical boosting algorithm, called AdaBoost, was proposed by Reference [23]. Because of its capability of generalization, fast performance, and low implementation complexity, boosting has become one of the most popular and effective classification tools [24]. The gradient boosting model proposed by Reference [25] is another widely used ensemble method for classification and regression problems. The gradient boosting algorithms use decision or regression trees as weak classifiers, and measure the error observed in each node and split node using a test function. Gradient boosting is similar to random forest in terms of combination of week tree leaners, but the tree in gradient boosting is fit on the residual of the former trees so that it can minimize the biases while random forest reduces variances [26].

On the other hand, due to the complicated structure of ensemble methods with multiple trees, their power of interpretability is weaker than algorithms with a single decision tree. However, even though decision-tree-based algorithms have better interpretability over other ML techniques, they still have limitations such as overfitting [27]. For example, if the training dataset is not large enough or includes some noise, the algorithms try to fit every single instance in the training set. As a result, the size of decision tree is relatively larger with meaningless branches and the overfitting problem leads to low interpretability. Thus, in order to have interpretability as well as high performance, shrinkage methods for ML models, such as discretization, are worthy for further research as a desirable property for interpretable models [28,29].

### 2.3. Discretization

Discretization, as one of the basic reduction techniques, has received attention because it helps decision trees to yield more compact, shorter, and more accurate results than the ones derived using numerical values [30]. Moreover, discretized attributes are easier to comprehend, employ, and describe for researchers [31]. 

Assume a classification problem with C target classes with a set of N instances and M attributes. A learning set $E=\left\{e_{1},\;e_{2},\;\ldots ,\;e_{N}\right\}$ consists of N instances. Each instance e∈E is described by M attributes $A_{1}\left(e\right),\;A_{2}\left(e\right),\;\ldots ,\;A_{M}\left(e\right)$, and labeled by a class c(e)∈C. A discretization algorithm partitions a continuous attribute $A_{i}$ into $k_{i}$ discrete and disjoint intervals as shown below:

$$D_{i}=\left\{\left[d_{0},d_{1}\right],\;\left(d_{1},d_{2}\right],\ldots ,\left(d_{k_{i}-1},d_{k_{i}}\right]\right\}$$

where $d_{0}$ and $d_{k_{i}}$ are the minimum and maximum values of $A_{i}$, respectively [32]. Finally, $P_{i}=\left\{d_{1},\;d_{2},\;\ldots ,\;d_{k_{i}-1}\right\}$ denotes the complete set of cut points for each continuous attribute i in M. The goal of discretization algorithms is to find the best $P_{i}$ for the target attribute i.

There are three different categories where discretization methods can be classified: global vs. local, supervised vs. unsupervised, and static vs. dynamic [33]. *Local* methods generate partitions that are applied to localized regions of the instance space while *global* methods, such as binning, independently produce a mesh over the entire n-dimensional continuous instance space. The mesh contains $\overset{M}{\underset{i=1}{\prod }}k_{i}$ regions, where $k_{i}$ is the number of partitions of the ith feature. *Unsupervised* discretization methods, such as equal width interval binning, do not use instance labels in the discretization process, while *supervised* discretization methods utilize the class labels. *Static* discretization methods perform determine the maximum number of intervals for each attribute independently, but *dynamic* methods conduct a search through the space of possible values for all attributes simultaneously to capture interdependencies. 

### 2.4. Related Work

Discretization has been proven to improve the performance as well as the interpretability of ML models, especially for decision tree models. Especially, discretization of multiple attributes can be considered as an optimization problem, which finds the best $P_{i}$ with consideration of interdependencies between attributes. The previous literature related to multivariate discretization with interpretable ML models is summarized in Table 1.

Reference [34] noted that C4.5’s performance is weaker in domains with a preponderance of continuous attributes than for learning tasks that have mainly discrete attributes. In order to address the weakness, a penalty inspired by the Minimum Description Length (MDL) principle was applied and it produced smaller DTs with higher accuracies with multi-interval splits. The results also showed that global discretization may degrade performance more as datasets become larger. Reference [35] focused on identifying the best combination of feature selection and discretization with four discretization methods: equal frequency binning (EFB), equal width binning (EWB), MDL, and ChiMerge. In this research, C4.5 was used for feature selection while SVM was used as a classifier.

Recently, as the size of data increases, finding the optimal discretization strategy with cut points is becoming extremely complicated. In order to solve the optimization problem, evolutionary multivariate discretizers (EMDs) have been studied for the discretization problem. Reference [32] proposed an evolutionary algorithm for learning decision rules with multivariate discretization called EDRL-MD (Evolutionary Decision Rule Learner with Multivariate Discretization). EDRL-MD consists of two steps: the simultaneous search for threshold values for all continuous attributes and the discovery of decision rules. Reference [36] proposed an evolutionary algorithm to construct a global discretization scheme for all continuous attributes simultaneously. The proposed algorithm was able to improve the accuracy of DTs and generate much simpler model. Reference [37] proposed an evolutionary algorithm to select a subset of cut points for multivariate discretization based on a wrapper fitness function. The algorithm was compared with different discretizers with C4.5 and Naive Bayes. Reference [38] proposed an evolutionary approach, which obtains a set of discretization schemes guiding the search by using a discretization criterion and the prediction accuracy of Naive Bayes. In Reference [39], classification error and number of cut points are simultaneously reduced by using evolutionary multi-objective optimization.

In addition to improved accuracy, discretization is likely to enhance interpretability, especially in combination with decision tree models [40]. For example, when applying discretization in C4.5, this benefit is clear, even when the continuous attributes are simply partitioned into ‘low’, ‘medium’, or ‘high’ values as shown in Figure 1b. Moreover, discretization can significantly increase the efficiency of decision tree induction by reducing the required sorting step for continuous attributes at each branch [41].

However, although the above-mentioned studies have proposed various discretization approaches, in practice, two issues persist. The first issue is the computational complexity. As the number of sensors increases with the stream of industrial IoT in manufacturing, the search space for global discretization has become voluminous. Accordingly, when the number of instances and continuous attributes increases, the chromosome structure for searching all possible cutting points may not be appropriate. 

Another issue is the lack of investigation on benefits of discretization under the limitation of the maximum tree depth. Although limiting the tree height for interpretability may affect the performance, the evolutionary approach for global discretization of a decision tree under the limitation of the maximum tree depth has been far less studied. Therefore, a new approach for learning interpretable models that are compact in size as well as sufficiently accurate is necessary to predict faults at the early stages and identify their root causes in an understandable form.

## 3. Proposed Approach

**Figure 2:**
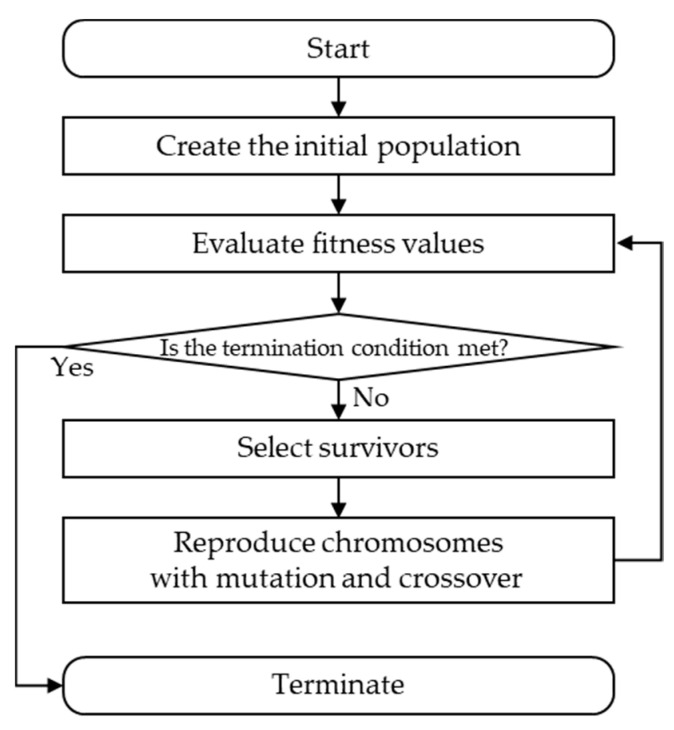
Overall framework of DIMPLED.

In this section, a novel evolutionary algorithm for global discretization called Decision tree Improved by Multiple sPLits with Evolutionary algorithm for Discretization (DIMPLED) is proposed. The proposed DIMPLED algorithm gradually improves the discretization strategy for better performance while maintaining the appropriate level of interpretability with a single decision tree. Also, in combination with *k*-means clustering for global discretization, DIMPLED allows a tree to have multiple splits that can be interpretable and meaningful for practitioners. The entire framework is first described, and then its detailed procedures are explained. The proposed DIMPLED framework can be summarized in Figure 2.

### 3.1. Chromosome Design

**Figure 3:**
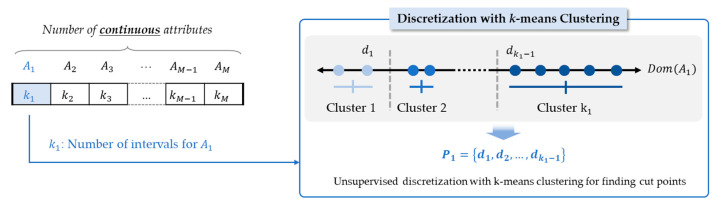
Chromosome representation.

Each chromosome consists of a given number of genes as shown in Figure 3. The length of a chromosome represents the number of continuous attributes that can be discretized. Each gene stores a discretization strategy $P_{i}$ that partitions a continuous attribute $A_{i}$ into the number of discrete intervals $k_{i}$, which is determined with consideration of the level of interpretability.

The initial population is generated by determining the number of classes for each continuous attribute randomly between 2 (not to be discretized, because binary split is the default) to the maximum number of intervals. The initial population with randomly generated chromosomes has been widely used in EMD, because it can cover the complete search space as much as possible and enhance the diversity as well [37,38,39]. For discretization of a continuous attribute with the given number of intervals in a chromosome, *k*-mean clustering algorithm partitions the values of continuous attributes into *k* clusters with the objective of making the clusters as separated as possible [42]. *k*-mean clustering has been used in unsupervised and global discretization to assist comprehension by grouping together multiple values of a continuous attribute [43]. In this study, the associated cost function is defined in terms of the distances between the cluster objects and the cluster center, and the objective is to find the best combination of $k_{i}$ intervals that maximizes the accuracy.

### 3.2. Reproduction

**Figure 4:**
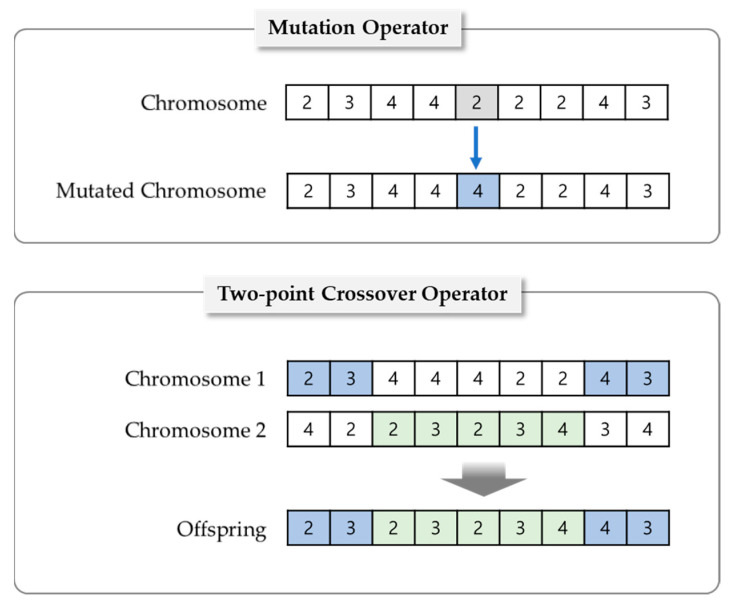
Illustration of mutation and crossover operations.

As shown in Figure 4, a set of new chromosomes for the next generation is generated by reproduction with two operators (mutation and crossover) based on the surviving chromosomes after selection. To make a change in a discretization strategy, the mutation operator randomly selects a gene in a single chromosome and reassigns it to another number of intervals. In the case of crossover, a two-point crossover operator is applied, and it changes only a certain part between two points.

### 3.3. Evaluation

**Figure 5:**
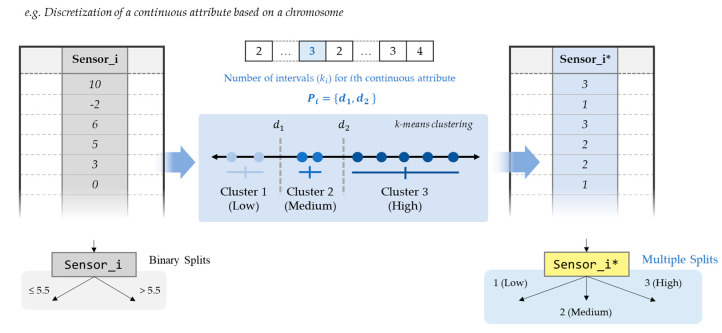
Evaluation of a chromosome with *k*-means clustering for discretization.

After the generation of chromosomes up to the given size of population, the chromosomes are evaluated by their accuracy with different discretization strategies. To calculate the accuracy of a chromosome, the continuous attributes in the training dataset are first discretized according to genes, which represent different number of intervals for attributes. If the number of intervals in a gene is greater than 2, values in the corresponding attribute are converted to discrete values (such as low, medium, and high), as shown in Figure 5. As a result of the global discretization with combination of C4.5, a smaller decision tree with multi-interval splits can be constructed, and it can be more accurate in some domains [34]. Also, by dividing the continuous values into interpretable intervals, discretization can improve the clarity of rule sets that are interpretable and meaningful to domain experts [44,45].

When making a set of multiple branches for an attribute, a decision tree may encounter missing classes at the bottom of the tree when the size of the training data is not sufficient [19]. In order to deal with those missing values, the missing class at the bottom is randomly labeled as one of classes as an interim measure, and the tree is reconstructed with the updated training data when the corresponding instances are supplemented.

### 3.4. Selection

In order to preserve the desirable characteristics of chromosomes for the next generations, the tournament selection selects surviving chromosomes and a fitness function is represented as the accuracy. The tournament selection has been widely used and implemented in evolutionary algorithms including EMD due to its lack of stochastic noise [38,46]. The tournament selection runs several tournaments among a set of chromosomes randomly selected from the population, and the winner of each tournament is elected for the next survivor. The termination criterion is the maximum number of generations.

## 4. Experimental Design

### 4.1. Data Description

To validate the performance of DIMPLED with real-world datasets from sensors, two classification datasets (CNC and Pasteurizer) of fault detection in manufacturing were used. The datasets were collected by Korea AI Manufacturing Platform (KAMP) from sensors on the shop floor, and they were pre-processed to eliminate noises and inadequate values [47]. The summary of the datasets is shown in Table 2. As shown in the table, the CNC dataset has a larger number of continuous attributes with a smaller number of instances than the Pasteurizer dataset. Attributes of two datasets were collected from sensors with binary quality labels (“Faulty” or “Normal”). The detailed descriptions about the datasets are presented in the following sections.

### 4.2. Computerized Numerical Control (CNC) Dataset

When processing jobs in CNC Machines, the precision of products varies according to various factors, such as the velocity of a certain axis and positions. Thus, predictive models are necessary to prevent expected faults and schedule maintenance for achieving higher productivity. The dataset was collected from sensors attached to CNC machines in a factory producing automotive parts. The detailed descriptions of attributes in the CNC dataset are shown in Table 3.

### 4.3. Pasteurizer Dataset

In the pasteurizing process, it is important to identify the factors that may affect the quality for final products, such as taste and flavor. Specifically, the temperature of pasteurizer is the key element for predicting the quality. In order to analyze the factors and predict the quality, the dataset was collected for 8.5 months from programmable logic controllers (PLCs) that the pasteurizers were equipped with and the database management system in a factory producing powdered dairy products. In the factory, two different pasteurizers (A and B) are used in parallel to accelerate the process and the dataset consists of the state and temperature of the two pasteurizers, and the quality of the final product. The state of the pasteurizer can be categorized into two values: 1 (RUN) and 0 (STOP). The detailed descriptions of attributes in the Pasteurizer dataset are summarized in Table 4.

## 5. Results and Discussions

This section presents the experimental results and compares the performance of the proposed DIMPLED algorithm to other tree-based algorithms, including ensemble learning methods, such as Random Forest, AdaBoost, and Gradient Boosting. The maximum tree height may impact how a tree-based algorithm attains interpretable structures, including the logic as well as the accuracy. Thus, in order to maintain the appropriate level of interpretability through tree-based models, the maximum tree height is determined as 3 based on the previous literature on tree-based algorithms [48,49]. Note that the level of interpretability of the DIMPLED can be also tuned by changing the maximum tree height. In a similar vein, the maximum number of intervals was limited to 4 for maintaining the interpretability. The detailed parameters for the algorithms are listed in Table 5. In the case of other tree-based algorithms, scikit-learn packages were used with the default setting, except for the maximum tree depth. The experiments were run on an Intel i9 10,900 3.7 GHz processor with 32 GB of RAM and GeForce RTX 2080 Ti.

To compare the performance of the tree-based algorithms, the performances obtained by the five algorithms are compared in terms of the average accuracy and interpretability using the CNC and Pasteurizer datasets described in Section 4. First, the average accuracy was calculated by the well-known tenfold cross-validation, which divides the dataset into 10 mutually exclusive and exhaustive partitions. In this paper, two datasets were partitioned using the stratified tenfold cross-validation. Also, the interpretability of the resulting trees was evaluated by splitting each dataset into two sets such that 70% of the data was used for training and 30% was used for testing.

### 5.1. Comparison Between Algorithms for Average Performance

To compare the performance of the tree-based algorithms, the average classification accuracy obtained by DIMPLED and the other algorithms are compared in Table 6. The table also includes the standard deviation for further comparisons. Among the tree-based algorithms, the gradient boosting algorithm outperformed the others in terms of the average accuracy and standard deviation for the two datasets. Also, in the case of the Pasteurizer dataset, the performances of some ensemble methods (Random Forest and AdaBoost) were significantly weakened compared to the result of the CNC dataset due to the limited tree depth. However, even though ensemble methods produced better performances than single-tree-based algorithms (C4.5, CART, and DIMPLED), the level of interpretability for ensemble models may not be appropriate for practitioners, because they have a lot of trees having different structures. In addition, decision trees generated by DIMPLED showed competitive performance compared to the widely-used ensemble methods in practice.

Furthermore, DIMPLED could produce significantly better performance than C4.5 and CART without loss of its interpretability. One possible explanation for this result is that the performance and generalizability of a single decision tree could be improved by having multiple splits with discretization.

### 5.2. Comparison Between Algorithms for Interpretability

**Figure 6:**
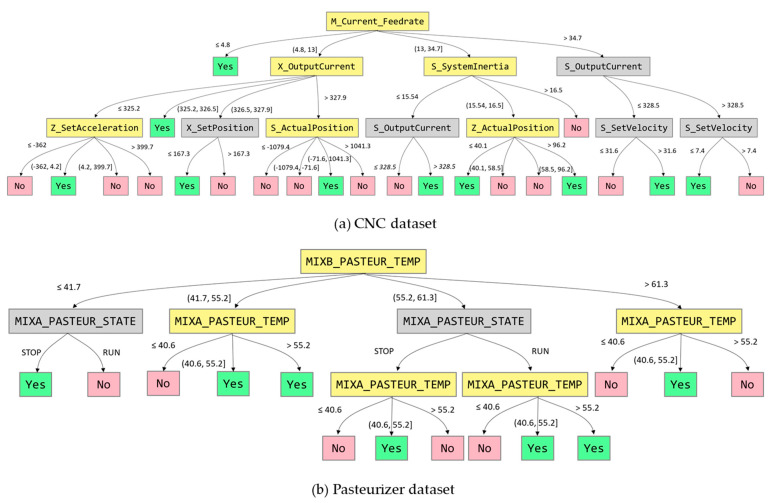
Illustration of two decision trees improved by DIMPLED. The decision trees contain discretized attributes highlighted in yellow, which have multiple branches.

**Figure 7:**
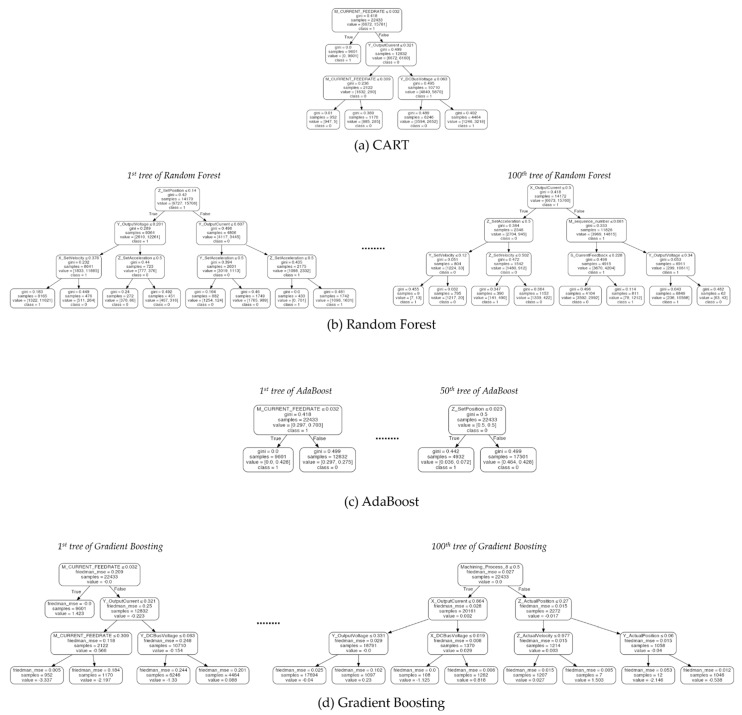
Illustration of decision trees generated by four tree-based algorithms (CART, Random Forest, AdaBoost, and Gradient Boosting) for the CNC dataset. Ensemble methods producing different decision trees are illustrated by visualizing only the first and last models.

**Figure 8:**
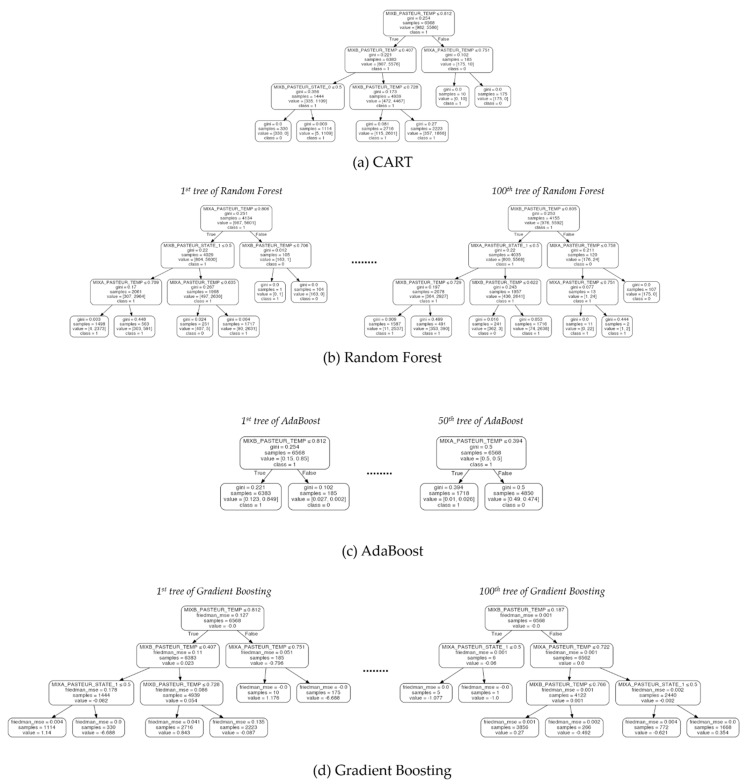
Illustration of decision trees generated by four tree-based algorithms (CART, Random Forest, AdaBoost, and Gradient Boosting) for the Pasteurizer dataset. Ensemble methods producing different decision trees are illustrated by visualizing only the first and last models.

To compare the interpretability of DIMPLED and the tree-based algorithms, each dataset was split into two sets: 70% of the data was used for training and 30% was used for testing. Based on the training and test datasets, tree-based models were generated, and their performances were compared in terms of the training and test accuracies, which are summarized in Table 7. In order to compare the interpretability of models in detail, the decision trees are depicted in Figure 6, Figure 7 and Figure 8.

In terms of the training and test accuracies, the results showed that gradient boosting algorithm outperformed the other tree-based algorithms. Similarly to the result of cross-validation, the benefits from combining multiple classifiers in Random Forest and Adaboost were not significant in the Pasteurizer dataset. However, in spite of its high performance, the interpretability of gradient boosting algorithm was significantly lower than the models based on a single decision tree as shown in Figure 6, Figure 7 and Figure 8. In the case of single decision trees, CART and DIMPLED are much easier to understand than the other algorithms, due to their simple and compact structures. 

Even though CART and DIMPLED have a similar power of interpretability, DIMPLED significantly outperformed CART in terms of both the training and test accuracies as shown in Table 6. Furthermore, DIMPLED could identify the root causes and their interdependencies as shown in Figure 6. For example, in the case of Pasteurizer dataset, when Pasteurizer B’s temperature was low-to-medium (between 41.7 and 55.2 °C) and Pasteurizer A’s temperature was over 40.6 °C, faulty products were observed in the training data. Also, in the case of CNC dataset, when the current feed rate was low-medium (between 4.8 and 13 mm/s) and the current of X output was low-to-medium (between 325.2 and 326.5 A), faulty products were observed in the training data.

In summary, the results demonstrate that DIMPLED can offer good interpretability compared with the other tree-based algorithms. In addition, DIMPLED appears to find an improved decision tree with the evolutionary process for global discretization, because the tree provides significantly better performance than does C4.5 and CART. Also, the model and its discretized attributes are completely transparent and interpretable, which can make the manufacturing systems more understandable, and thus reliable to human operators.

## 6. Conclusions and Future Work

This paper addressed the classification model for fault detection in manufacturing. In order to identify the root causes on the shop floor, interpretable ML models that can provide insights as an understandable form are crucial to improving the product quality. However, due to the recent trend of IoT, the number of sensors is exploding, and thus the generation of ML models with high-performance and appropriate level of interpretability is becoming more complicated. To deal with the fault detection problem effectively, a new approach called DIMPLED for evolutionary discretization is proposed. The proposed DIMPLED algorithm improves the structure of a single decision tree by evolving discretization strategies so that it enables the tree to have multiple splits. The experimental results with two datasets in manufacturing show that the decision tree improved by DIMPLED outperformed the performance of C4.5 and CART used widely in practice and it was competitive compared to the ensemble methods, which require multiple decision trees. Even though the ensemble methods could produce slightly better performances, the proposed DIMPLED has more interpretable structure while maintaining the appropriate performance level.

The major contribution of this paper is the development of a new approach for capturing insights with the appropriate level of interpretability. To improve the accuracy with the limited tree height, the proposed DIMPLED enables a tree to have multiple splits with automated discovery process of the best discretization strategy. Also, by the benefit of the reduced set of rules from a simple decision tree, the models generated by DIMPLED have the capability for fault prediction in real-time. Moreover, based on the improved tree, human operators can improve the product quality by identifying the root causes as a set of IF-THEN rules and thus DIMPLED is expected to be utilized to various fault detection problems without the black-box issue.

Future work can proceed in several directions. First, considerations of other interpretable algorithms are interesting and worthy for investigation. Additionally, applications of DIMPLED to other types of classification problems can be studied. Finally, feature extraction techniques can be supplemented to DIMPLED for consideration of their potential effects.

## Figures and Tables

**Table 1:** Overview of research related to multivariate discretization with interpretable ML models.

Discretization Algorithm	Classifier	Reference
Minimum Description Length (MDL)	C4.5	[34]
Equal Frequency Binning (EFB), Equal Width Binning (EWB), MDL, and ChiMerge	C4.5 and SVM	[35]
Evolutionary Decision Rule Learner with Multivariate Discretization (EDRL-MD)	C4.5	[32]
Evolution-Strategies-based discretization Algorithms	ID3, C4.5, and C4.5-rules	[36]
Evolutionary algorithm for multivariate discretization based on a wrapper fitness function	C4.5 and Naive Bayes	[37]
Multivariate discretization algorithm (multiCAIM) based on NSGA-II	C4.5, Naive Bayes, and k-nearest neighbors (KNN)	[38]
Multivariate Evolutionary Multi-Objective Discretization (MEMOD)	C4.5	[39]

**Table 2:** Summary of two datasets (CNC and Pasteurizer).

Dataset	Number of Instances	Class Distribution (Faulty/Normal)	Number of Categorical Attributes	Number of Continuous Attributes
CNC	32,048	22,645/9403	2	46
Pasteurizer	210,794	77,784/133,010	2	2

**Table 3:** Description of attributes in CNC dataset.

Source (Number of Attributes)	Attribute Name (Unit of Continuous Attribute or Description of Categorical Attribute)
*X*-axis	X_ActualPosition (mm), X_ActualVelocity (mm/s), X_ActualAcceleration (mm/s/s), X_SetPosition (mm), X_SetVelocity (mm/s), X_SetAcceleration (mm/s/s), X_CurrentFeedback (A), X_DCBusVoltage (V), X_OutputCurrent (A), X_OutputVoltage (V), X_OutputPower (kw)
(11)	
*Y*-axis	Y_ActualPosition (mm), Y_ActualVelocity (mm/s), Y_ActualAcceleration (mm/s/s), Y_SetPosition (mm), Y_SetVelocity (mm/s), Y_SetAcceleration (mm/s/s), Y_CurrentFeedback (A), Y_DCBusVoltage (V), Y_OutputCurrent (A), Y_OutputVoltage (V), Y_OutputPower (kw)
(11)	
*Z*-axis	Z_ActualPosition (mm), Z_ActualVelocity (mm/s), Z_ActualAcceleration (mm/s/s), Z_SetPosition (mm), Z_SetVelocity (mm/s), Z_SetAcceleration (mm/s/s), Z_CurrentFeedback (A), Z_DCBusVoltage (V), Z_OutputCurrent (A), Z_OutputVoltage (V)
(10)	
Spin	S_ActualPosition (mm), S_ActualVelocity (mm/s), S_ActualAcceleration (mm/s/s), S_SetPosition (mm), S_SetVelocity (mm/s), S_SetAcceleration (mm/s/s), S_CurrentFeedback (A), S_DCBusVoltage (V), S_OutputCurrent (A), S_OutputVoltage (V), S_OutputPower (kw), S_SystemInertia (kg·m^2^)
(12)	
Others	M_CURRENT_PROGRAM_NUMBER (3 categorical values), M_sequence_number (sequence number), M_CURRENT_FEEDRATE (mm/s), Machining_Process (9 categorical values)
(4)	

**Table 4:** Description of attributes in Pasteurizer dataset.

Source (Number of Attributes)	Attribute Name (Unit of Continuous Attribute or Description of Categorical Attribute)
Pasteurizer A (2)	MIXA_PASTEUR_TEMP (°C), MIXA_PASTEUR_STATE (2 categorical values)
Pasteurizer B (2)	MIXB_PASTEUR_TEMP (°C), MIXB_PASTEUR_STATE (2 categorical values)

**Table 5:** Parameters for DIMPLED and other tree-based algorithms.

Algorithm	Parameter	Value
DIMPLED	Population size	500
	Number of generations	20
	Mutation rate	0.3
	Crossover rate	0.3
	Tournament size	5
	Survivor size	50
	Maximum number of intervals	4
AdaBoost	Number of trees	50
Random Forest	Number of trees	100
Gradient Boosting	Number of trees	100

**Table 6:** Average test performances of DIMPLED and tree-based algorithms for CNC and Pasteurizer datasets in cross-validation.

	CNC					Pasteurizer				
	Accuracy		Avg. Precision	Avg. Recall	Avg. F_1_	Accuracy		Avg. Precision	Avg. Recall	Avg. F_1_
	Avg.	S.D.				Avg.	S.D.			
C4.5	66.66	±19.99	0.8078	0.7439	0.6994	91.01	±0.85	0.9211	0.9779	0.9486
CART	75.52	±15.95	0.8276	0.836	0.818	92.77	±0.74	0.922	0.9995	0.9592
Random Forest	81.79	±17.09	0.8766	0.8741	0.8662	92.93	±0.81	0.9232	1.0	0.9601
AdaBoost	80.62	±17.35	0.8919	0.8226	0.8409	91.04	±0.88	0.924	0.9746	0.9486
Gradient Boosting	87.03	±12.56	0.9287	0.8946	0.9008	99.7	±0.24	0.9979	0.9985	0.9982
DIMPLED	84.81	±10.43	0.8862	0.9118	0.8956	95.49	±5.18	0.9861	0.9607	0.9722

**Table 7:** Training and test performances of DIMPLED and tree-based algorithms for CNC and Pasteurizer datasets.

	CNC					Pasteurizer				
	Train Accr.	Test Accr.	Precision	Recall	F_1_	Train Accr.	Test Accr.	Precision	Recall	F_1_
C4.5	75.11	74.63	1.0	0.6457	0.7847	90.68	91.23	0.9228	0.9782	0.9497
CART	81.33	81.2	0.9195	0.8981	0.8602	92.74	93.0	0.9239	0.9996	0.9603
Random Forest	90.58	90.71	0.9286	0.9427	0.9356	93.38	93.64	0.9301	1.0	0.9638
AdaBoost	92.99	93.39	0.9513	0.9566	0.9539	90.64	91.55	0.9227	0.9824	0.9516
Gradient Boosting	96.56	96.71	0.9773	0.9799	0.9771	99.92	99.47	0.9962	0.9945	0.9969
DIMPLED	91.19	91.89	0.9421	0.9448	0.9434	98.11	98.05	0.9874	0.9895	0.9885
